# Renoprotective effects of a novel cMet agonistic antibody on kidney fibrosis

**DOI:** 10.1038/s41598-019-49756-z

**Published:** 2019-09-17

**Authors:** Yong Chul Kim, Junghun Lee, Jung Nam An, Jin Hyuk Kim, Young-Wook Choi, Lilin Li, Sang Ho Kwon, Mi-Young Lee, Boeun Lee, Jae-Gyun Jeong, Seung-Shin Yu, Chun Soo Lim, Yon Su Kim, Sunyoung Kim, Seung Hee Yang, Jung Pyo Lee

**Affiliations:** 1 Department of Internal Medicine, Seoul National University Hospital, Seoul, Korea; 2 R&D Center for Innovative Medicines, Helixmith Co., Ltd., Seoul, Korea; 3 Department of Internal Medicine, Seoul National University Boramae Medical Center, Seoul, Korea; 4 Department of Internal Medicine, Seoul National University College of Medicine, Seoul, Korea; 5 Department of Medical Science, Seoul National University College of Medicine, Seoul, Korea; 6 Kidney Research Institute, Seoul National University College of Medicine, Seoul, Korea; 7 Seoul National University Biomedical Research Institute, Seoul, Korea

**Keywords:** Pharmaceutics, Chronic kidney disease

## Abstract

Hepatocyte growth factor (HGF) and its receptor, cMet, activate biological pathways necessary for repair and regeneration following kidney injury. Because HGF is a highly unstable molecule in its biologically active form, we asked whether a monoclonal antibody (Ab) that displays full agonist activity at the receptor could protect the kidney from fibrosis. We attempted to determine whether the cMet agonistic Ab might reduce fibrosis, the final common pathway for chronic kidney diseases (CKD). A mouse model of kidney fibrosis disease induced by unilateral ureteral obstruction was introduced and subsequently validated with primary cultured human proximal tubular epithelial cells (PTECs). In kidney biopsy specimens from patients with CKD, cMet immunohistochemistry staining showed a remarkable increase compared with patients with normal renal functions. cMet Ab treatment significantly increased the levels of phospho-cMet and abrogated the protein expression of fibrosis markers such as fibronectin, collagen 1, and αSMA as well as Bax2, which is a marker of apoptosis triggered by recombinant TGF-β1 in PTECs. Remarkably, injections of cMet Ab significantly prevented kidney fibrosis in obstructed kidneys as quantified by Masson trichrome staining. Consistent with these data, cMet Ab treatment decreased the expression of fibrosis markers, such as collagen1 and αSMA, whereas the expression of E-cadherin, which is a cell-cell adhesion molecule, was restored. In conclusion, cMet-mediated signaling may play a considerable role in kidney fibrosis. Additionally, the cMet agonistic Ab may be a valuable substitute for HGF because it is more easily available in a biologically active, stable, and purified form.

## Introduction

Renal fibrosis, especially tubulointerstitial fibrosis (TIF) is a final clinical outcome of chronic kidney disease (CKD) that eventually progress to end-stage renal disease (ESRD) regardless of the diverse underlying causes^1–3^. Studies in both experimental animal models and human subjects show that tubulointerstitial damage results in the deterioration of renal function^4^. Although many investigational advances in understanding the molecular mechanisms of TIF has been achieved over recent years, potential therapeutic agents that inhibit or even reverse kidney fibrosis are needed.

cMet, the transmembrane tyrosine kinase receptor for hepatocyte growth factor (HGF), is critically involved in cell survival by activating signaling pathways, resulting in cell growth, regeneration, and the survival of cells and tissues^5–9^. These distinctive biologic property have generated great experimental interest that HGF/Met pathway could be a potential therapeutic alternatives for treating renal disease. There is several experimental evidence that indicates the anti-fibrotic effect of the HGF/Met pathway because administration of recombinant HGF protein or the HGF gene showed marked improvement of TIF^10–16^. We also recently demonstrated that activating the HGF/Met pathway ameliorates fibrosis in primary cultured glomerular endothelial cells^17^.

Despite recent studies supporting the definitive role of HGF in protecting kidney fibrosis, there are several limitations in terms of using HGF as a drug. HGF is difficult to purify in its heterodimeric biologically active form and is highly unstable^18^; it has a short half-life. To circumvent these shortcomings of HGF, we developed a monoclonal antibody (mAb) that could activate the cMet receptor and trigger the downstream pathways promoted by HGF.

In this study, we have elucidated that the cMet agonistic Ab efficiently protects against kidney fibrosis not only in *in vitro* models with primary cultured human proximal tubular epithelial cells (PTECs) but also in *in vivo* unilateral ureteral obstruction (UUO) kidney fibrosis models. This novel monoclonal cMet agonistic antibody can be a valuable substitute for the natural ligand HGF and could be easily reproduced in a biologically active, highly stable, and purified form.

## Results

### cMet expression in normal human kidney tissue

**Figure 1:**
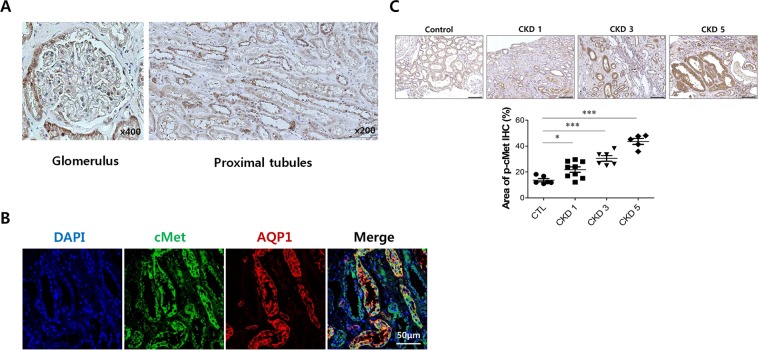
cMet expression in normal human kidney tissue. (**A**) Representative images of IHC staining for cMet in the glomerulus and tubulo-interstitium from normal human kidney tissue. Original magnification: X400 (Left), X200 (Right). Scale bar, 100 µm. **(B)** Representative confocal microscopy images of human kidney biopsy samples from human normal kidney tissues; the samples were costained for cMet (green), aquaporin-1 (red) and DAPI (blue). Original magnification: X200. **(C)** Representative images of phospho-cMet expression in IgAN patients. Original magnification: X200. Scale bar, 100 µm. Symbols represent individual data points, horizontal bars indicate the mean, and error bars indicate SEM (*p < 0.05, ***p < 0.001).

We found that proximal tubular epithelial cells (PTECs) expressing cMet were abundant in the healthy glomerulus (Fig. 1A,B). Next, we used immunofluorescence staining with a phosphorylation-specific cMet Ab to investigate whether the HGF/Met pathway is involved in kidney fibrosis in patients with IgA nephropathy (IgAN), which is one of most common chronic kidney diseases. As shown in Fig. 1C, assessment of the levels of active cMet in the tubulointerstitial area revealed that cMet was activated in patients with IgAN, and the expression increased as kidney function deteriorated (Control: 13.7 ± 1.3%, CKD stage 1: 21.8 ± 2.1%, CKD stage 3: 30.5 ± 2.2%, CKD stage 5: 43.6 ± 2.3%, P < 0.0001). The baseline characteristics of IgAN patients and healthy controls are listed in Supplementary Table S1.

### Effect of cMet agonistic antibody in human umbilical vein endothelial cells (HUVECs)

We developed an agonistic antibody that could act in a similar manner to HGF in this study to investigate whether this antibody could be effective in disease models.

**Figure 2:**
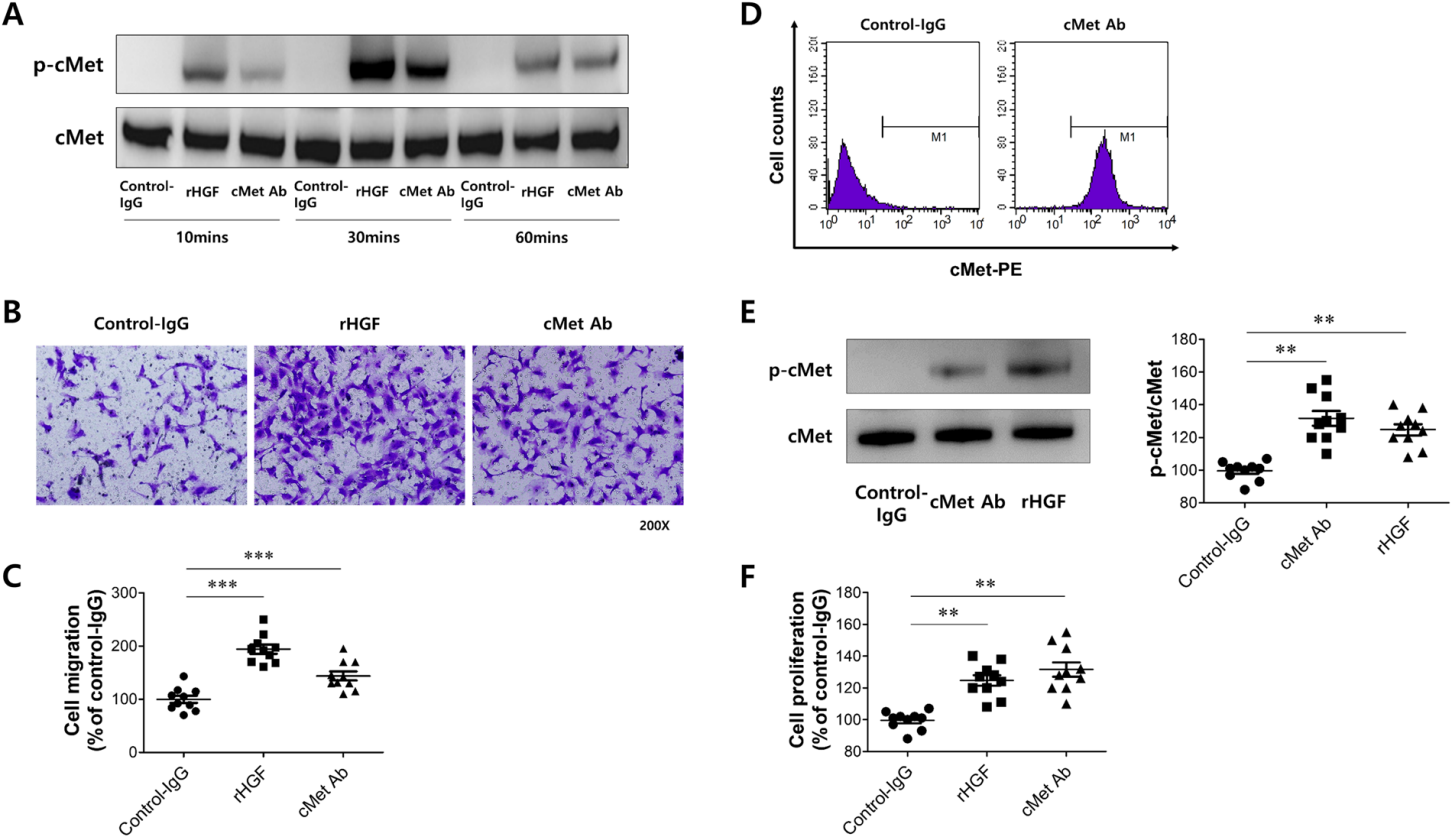
Effect of cMet agonistic antibody on human umbilical vein endothelial cells (HUVECs). (**A)** cMet Ab-mediated activation of the cMet receptor. **(B)** Representative image of the HUVEC migration assay. **(C)** Quantification of (**B**). **(D)** Analyzing the biological activity of the cMet Ab in A549 cells. **(E)** cMet Ab-mediated activation of the cMet receptor. **(F)** Representative image of cell proliferation assay in A549 cells.

For antibody screening and development, ScFv specific to human cMet was selected by the phage display method as described in the Methods section. Then, cMet Ab with IgG1 format was generated based on the selected ScFv antibody. This cMet Ab binds to both human and mouse cMet (Table. S2 in the Supplementary Information) and target specificity of cMet Ab was confirmed by microarray test (Fig. S1 in the Supplementary Information), suggesting cMet Ab binds to cMet in a highly specific manner. A preliminary study of pharmacokinetics suggested the half-life of this antibody was approximately 3 days (data not shown). In addition, *in vivo* biodistribution patterns suggested that the antibody was significantly located in the liver one hour post injection. It was also detected in the spleen, kidney, lung, and heart, respectively (Fig. S2 in the Supplementary Information). To investigate the biological activity of cMet Ab, we observed that vascular endothelial cell migration, which is typically induced by HGF, was activated. First, HUVECs were treated with cMet Ab to analyze whether cMet, the receptor for HGF, was activated. HGF was used as a control treatment for comparison. As shown in Fig. 2A, when cells were treated with cMet Ab, cMet was activated at 10 minutes after Ab treatment and peaked at 30 minutes. The activation of cMet by cMet Ab was significantly increased at 60 minutes compared to the levels in the control IgG-treated group, and such kinetics were similar to those in HGF-treated cells. Next, we examined whether the cMet Ab promotes HUVEC migration. After HUVECs were treated with the cMet Ab in a Boyden chamber, the number of migratory cells was quantified by staining the cells with crystal violet (Fig. 2B). As shown in Fig. 2C, HUVEC cell migration was significantly increased after HGF treatment as observed in previous studies. When the cells were treated with cMet Ab, HUVEC migration was significantly increased compared to that of the control-IgG treatment group. Based on these data, it was confirmed that the cMet Ab can activate the cMet receptor in a manner similar to that of HGF and promote HUVEC migration.

Another major biological function of HGF is its promotion of cell proliferation. A549 cells were used to determine whether the cMet Ab enhanced cell proliferation in a manner similar to that of HGF. First, fluorescence-activated cell sorting (FACS) was performed to determine whether cMet in A549 cells could be properly detected and activated by the cMet Ab. A549 cells were treated with cMet Ab and reacted with a secondary antibody recognizing the Fc of the Ab to detect bound cMet Ab via FACS. A significant shift in the FACS data distribution was observed in the cMet Ab-treated control group compared to the group treated with control antibody (Fig. 2D). To investigate whether cMet Ab activates cMet, A549 cells were treated with cMet Ab and assessed via western blot. The activity of cMet was increased in the cMet Ab-treated group compared to the control IgG-treated group (similar to the HUVEC data), and this increased activity was similar to that of the HGF-treated group (Fig. 2E). Finally, A549 cells were treated with HGF and cMet Ab and subjected to a proliferation assay. As shown in Fig. 2F, significant cell proliferation was observed in the group treated with cMet Ab. These results suggest that cMet Ab can bind to cMet and activate this receptor; furthermore, the proliferation of A549 cells treated with cMet Ab was similar to that of cells treated with HGF.

### *In vivo* validation: safety test in immune cells

**Figure 3:**
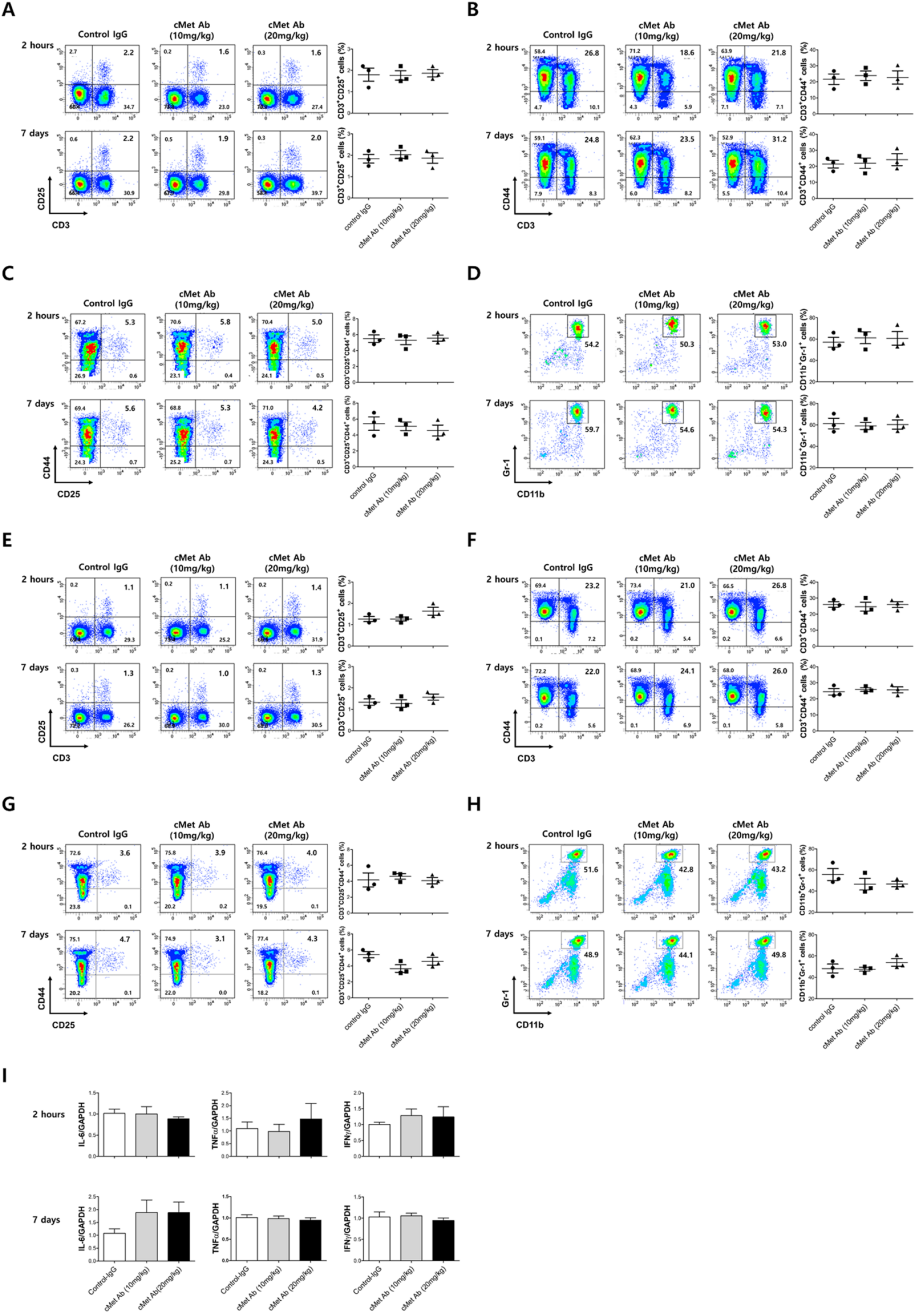
Injection of cMet Agonistic antibody in mice did not produce changes in the immune cell profile. After C57BL/6J wild-type mice were treated with cMet agonistic Ab (10 mg/kg, 20 mg/kg) or control IgG, their peripheral blood (Figure A-D) or spleens (Figure E-H) were harvested 2 hours later. Also we injected cMet agonistic Ab or control IgG under the same schedule as UUO experiment and their peripheral blood/spleens were harvested 7 days later. The different immune cell populations were enumerated by flow cytometry or an automated hematology analyzer. In the spleen cell suspensions, cMet treatment did not lead to obvious changes in the percentages of the CD3^+^ and CD25+ regulatory T cell populations **(A**,**E)**, CD3+CCD44+ effector T cell populations **(B**,**F)**, CD3+CCD25+CD44+ effector T cell populations **(C**,**G)**, or CD11b+Gr-1+ myeloid (nonlymphoid) cell populations **(D**,**H)** compared with those in the control IgG treatment group. **(I)** Pro-inflammatory cytokines expression (IL-6, TNFα, IFNγ) didn’t show difference between the spleen cells of control IgG or cMet Ab injected mice.

To evaluate whether the novel cMet Ab is a biologically safe *in vivo*, a safety test was performed. After C57BL/6J wild-type mice were intravenously injected intravenously with cMet Ab (10 mg/kg, 20 mg/kg) or isotype control IgG, their peripheral blood and spleens were harvested 2 hours and 7days later. The immune cells present were enumerated by flow cytometry or an automated hematology analyzer. In the peripheral blood cell suspensions, cMet treatment did not lead to obvious changes in the percentages of CD3^+^CD25^+^ regulatory T cell populations (Control IgG vs cMet Ab (10 mg/kg) vs cMet Ab (20 mg/kg), 2 hours: 1.80 ± 0.53% vs 1.77 ± 0.38% vs 1.87 ± 0.31%, 7days: 1.83 ± 0.35% vs 2.03 ± 0.32% vs 1.87 ± 0.41%, Fig. 3A), CD3^+^CD44^+^ effector T cell populations (2 hours: 21.6 ± 5.7% vs 23.8 ± 5.1% vs 22.8 ± 7.2%, 7days: 21.4 ± 4.1% vs 21.9 ± 5.5% vs 24.1 ± 6.4%, Fig. 3B, CD3^+^CD25^+^CD44^+^ effector T cell populations (2 hours: 5.50 ± 0.82% vs 5.30 ± 0.95% vs 5.57 ± 0.67%, 7days: 5.43 ± 1.46% vs 5.10 ± 0.92% vs 4.57 ± 1.19%, Fig. 3C, or CD11b^+^Gr-1^+^ myeloid (nonlymphoid) cell populations (2 hours: 57.1 ± 8.0% vs 61.2 ± 9.6% vs 60.8 ± 11.4%, 7days: 61.3 ± 8.6% vs 59.1 ± 5.6% vs 60.4 ± 7.5%, Fig. 3D). Similarly, in the spleen cell suspensions, cMet treatment did not induce any significant changes in the percentages of immune cells (Fig. 3E–H). Quantitative PCR data showed that pro-inflammatory cytokines expressions such as IL-6, TNFα, and IFNγ were comparable between the splenocytes of control IgG or cMet Ab injected mice (Fig. 3I). These data suggest that the newly developed cMet Ab has minimal or no immunogenicity, which could lead to the formation of antidrug antibodies (ADAs).

### Effects of cMet agonistic Ab on kidney fibrosis in the UUO model

**Figure 4:**
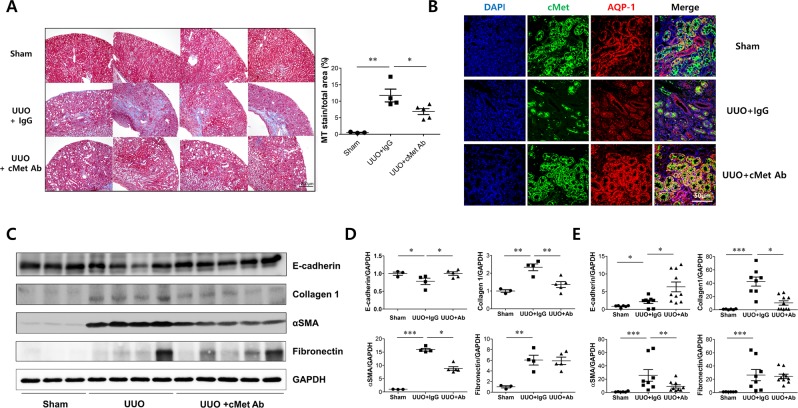
cMet agonistic Ab and kidney fibrosis in a unilateral ureteral obstruction (UUO) mouse model. **(A)** Representative images of kidney sections stained with Masson’s trichrome stain in IgG control- or cMet Ab-treated mice at day 14 after UUO. Original magnification: X20. Scale bar, 200 µm. Symbols represent individual data points, horizontal bars indicate the mean, and error bars indicate SEM. **(B)** cMet expression in proximal tubular cells in n IgG control- or cMet Ab-treated mice at day 14 after UUO. DAPI (blue), cMet (green), and aquaporin-1 (red).Original magnification: X400. Scale bar, 50 µm. **(C**,**D)** The expression levels of fibronectin and collagen 1 were increased and those of E-cadherin were decreased in the cMet group, and treatment with the cMet agonistic Ab prevented fibrosis as indicated by western blot. (**E**) Quantitative RT-PCR analyses of the indicated mRNA transcript showed similar expression patterns as those observed by western blot. The combined data represent at least 2 independent experiments with similar results. Symbols represent individual data points, horizontal bars indicate the mean, and error bars indicate SEM (*p < 0.05, **p < 0.01, ***p < 0.001).

To demonstrate the antifibrotic efficacy of the cMet agonistic Ab, we first conducted an *in vivo* study with a UUO mouse model and quantitated kidney fibrosis by Masson’s trichrome staining. Two weeks after UUO, kidney fibrosis was extensive; however, cMet Ab-treated UUO mice exhibited reduced kidney fibrosis (MT stain/total area, Sham: 0.53 ± 0.06%, UUO: 12.08 ± 0.87%, UUO .8cMet Ab: 6.57 ± 0.48%; Fig. 4A). Confocal image showed that cMet expression was increased in proximal tubular cells in sham and UUO mice kidney, this suggests that cMet Ab could act as an agonistic Ab directly to the proximal tubules of UUO mice (Fig. 4B). To further test whether cMet Ab administration would reduce the development of kidney fibrosis, we performed western blot analyses. As shown in Fig. 4C,D, there was a marked increase in the expression of collagen 1 and αSMA in the obstructed kidney, suggesting that kidney fibrosis was properly induced. In addition, the expression of these two proteins was decreased after administration of cMet Ab. In the real- time PCR assay, collagen 1 and αSMA levels were increased after UUO, and these increases were less pronounced after cMet Ab treatment (Fig. 4E). Collectively, these data provide experimental evidence that pharmacological activation of cMet with an agonistic Ab might represent a novel therapeutic strategy in treating kidney fibrosis.

### Effect of cMet agonistic antibody on primary cultured human proximal tubular epithelial cells (PTECs)

**Figure 5:**
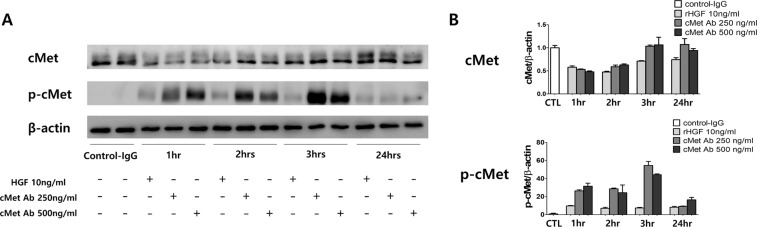
Effect of the cMet agonistic antibody on primary cultured human proximal tubular epithelial cells (PTECs). (**A)** Representative western blot analysis of primary cultured human PTECs after treatment with rHGF (10 ng/ml) or cMet Ab (250, 500 ng/ml) for various time periods. **(B)** The expression of phosphorylated cMet was increased at 1 hour and 3 hours after treatment with rHGF or cMet Ab.

To determine the *in vitro* efficacy of the cMet agonistic Ab, we used primary cultured human PTECs. A representative western blot analysis was performed with primary cultured human PTECs after treatment with rHGF (10 ng/ml) or cMet Ab (250, 500 ng/ml) in various time periods. (Fig. 5A,B) The expression of phosphorylated cMet was appropriately increased at 1 hour and 3 hours after treatment with rHGF or cMet Ab.

**Figure 6:**
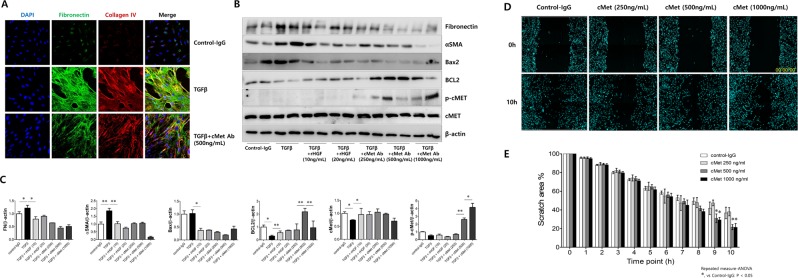
Effect of cMet agonistic antibody on kidney fibrosis and wound healing in primary cultured human proximal tubular epithelial cells (PTECs) *in vitro*. **(A)** Representative confocal images of primary cultured human PTECs costained for DAPI (blue), fibronectin (green), and collagen IV (red). Fibrosis was induced with TGFβ, and treatment with the cMet agonistic Ab (500 ng/ml) indicated improvements in fibrosis. Original magnification: X200. **(B**,**C)** In the presence or absence of either rHGF (10, 20 ng/ml) or cMet Ab (250, 500, 1000 ng/ml) for 30 minutes, rTGFβ-stimulated PTECs exhibited significantly increased protein levels of fibronectin, αSMA and Bax2. **(D**,**E)** The migratory capacity of PTECs was assessed using a scratch wound healing assay and observing cell movement at 0 and 10 hours after scratching. There was a significant difference in the migratory potential of cells in the high-dose cMet Ab group compared to that in the control groups. Data represent the mean ± SD of three independent experiments. *P < 0.05, **P < 0.01 versus control group.

Next, to assess whether exogenous cMet Ab prevents kidney cell fibrosis, primary cultured human PTECs were treated with rTGFβ (to induce fibrosis) in the presence or absence of cMet Ab. Confocal images revealed that rTGFβ induced the expression of fibronectin and collagen IV, and cMet Ab blocked this expression (Fig. 6A). Western blot analysis also demonstrated that rTGFβ increased fibronectin and αSMA protein expression, which are markers of fibrosis, and Bax2, an apoptosis marker. Cells cotreated with cMet Ab exhibited decreased expression of fibronectin, αSMA, and Bax2 and increased BCL2 and phospho-cMet expression (Fig. 6B,C).

The wound healing assay is a laboratory procedure used to study cell migration and cell-to-cell interaction. PTECs were plated in 48-well plates after a 2nd passage and cultured to form a monolayer. After 6 hours of serum starvation, the monolayer was scratched with a p20 pipette tip through the center of the well, and the cell growth into the scratched area was monitored by immunofluorescence and bright-field microscopy at 0 and 10 hours after scratching (Fig. 6D). During the 10-hour period, the cells of cMet Ab-treated group showed markedly faster cell migration than untreated group (Fig. 6E). Taken together, these data clearly suggest that the cMet agonistic Ab blocks fibrosis and apoptosis and promotes wound healing *in vitro*.

## Discussion

Kidney fibrosis, mainly characterized by TIF, is a common pathological outcome of CKD, regardless of the etiology^2,19,20^. Progressive CKD often results in excessive accumulation of extracellular matrix, which leads to complete destruction of the kidney and end-stage renal failure that requires either dialysis or kidney transplantation^21^. While extensive experimental studies have provided comprehensive understanding^22,23^ and promising therapeutic options^24,25^ for treating CKD, identifying antifibrotic treatments for kidney remains a major challenge in nephrology^26^.

The present investigation demonstrates that a monoclonal antibody that activates the HGF/Met pathway can protect against kidney fibrosis in primary cultured human PTECs *in vitro* and in a UUO mouse model *in vivo*. To the best of our knowledge, this is the first study to demonstrate that our cMet agonistic monoclonal antibody could treat renal disease, especially as it relates to kidney fibrosis. More importantly, we validated the efficacy of this novel drug in three different cell types (HUVECs, A549 cells, primary cultured human PTECs). Additionally, immunogenicity biosafety tests relating to various immune cells were performed.

HGF is a growth factor that exhibits a variety of biological functions and activates many types of downstream signaling pathways through the cMet receptor^27^. In particular, its expression is increased when the tissue is damaged, thereby contributing to tissue repair and recovery^28–30^. Because of the biological characteristics of HGF-mediated activity^31^, many attempts have been made to apply HGF to disease. HGF has been shown to exert antifibrotic activities in various organs, including the liver, lung and kidney^5,32,33^. Although HGF is well known for its antifibrotic potential, its short half-life is an obvious hurdle for the development of efficient therapeutics using or targeting this growth factor. A previous report calculated the half-life of recombinant HGF protein at 2.4 minutes^34^, which makes it difficult to achieve a specific therapeutic dose for HGF.

Antibodies may be a useful platform to overcome the short half-lives of growth factors because antibodies usually exhibit longer half-lives than recombinant proteins^35^. By designing agonistic or receptor-activating antibodies, the activities of growth factors could be mimicked, as shown in various reports^36,37^.

In this study, we designed a novel antibody that can activate the cMet receptor to mimic HGF protein. We found that this antibody could induce phosphorylation of the cMet receptor and activate biological functions such as cell migration and proliferation, which are hallmarks of HGF protein activity^27^. Additionally, we found that this antibody could exert therapeutic effects in the UUO mouse model, suggesting that the cMet agonistic antibody can be used to treat kidney disease.

Unlike the general PK profile of conventional antibodies, the cMet Ab used in this study showed a relatively rapid clearance rate when compared to that of a commercialized antibody (data not shown). Further analysis showed that the stability of this antibody in plasma was comparable to that of Herceptin. Additional investigations to elucidate the possible mechanism of this rapid clearance rate are currently underway.

Recently, monoclonal antibody-based therapy has been established as one of the most powerful therapeutic entities for several human diseases. Antibody-based targeting of cMet offers promising outcomes over recombinant HGF or HGF gene therapies. The first challenge comes from the rapid clearance of rHGF in the peripheral circulation, which inevitably requires repetitive injections at short intervals. In previous studies, recombinant human HGF (rhHGF) was administered subcutaneously every 12 hours for 4^38^ to 11^39^ consecutive days in a UUO model. To circumvent this problem, gene therapy with a plasmid containing the HGF coding region was utilized to maintain the plasma HGF concentration with a single injection per week^12,16,40,41^. Nevertheless, large-scale manufacturing of plasmids encoding HGF at high purity and concentrations is difficult. On the other hand, we demonstrated here that in UUO model mice, intravenous injection of cMet Ab (20 mg/kg) once every 2 to 4 days led to prominent amelioration of kidney fibrosis. In addition, we have successfully established a process to produce cMet Ab with high purity on a large scale.

In conclusion, we demonstrated that the HGF/Met pathway plays a dominant role in kidney fibrosis. We also showed that agonistic antibodies targeting cMet can be generated and used as a therapeutic agent for treating kidney fibrosis *in vivo* and *in vitro*. Taken together, these data provide support that cMet agonistic Ab is a novel therapeutic approach against kidney fibrosis and is a valuable substitute for HGF owing to its better availability in a biologically active, stable, and purified form.

## Methods

### Generation of cMet-specific antibody

In order to generate cMet-specific antibody, biopanning process was performed with single chain antibody (ScFv) phage display library. Initially, phages showing anti-human Fc ScFv and other non-specific binding were removed in prepared the phage.

Next, 96 well NUNC-IMMUNO plate (Nunc, Denmark) coated with cMet-Fc (disclose upon request) was blocked with 2% skim milk/PBS at room temperature for 2 h, and then 3 × 10^11^ PFU of the prepared phage was inoculated to each well at room temperature for 1 h. The wells were washed 5 times in PBS with/without 0.1% Tween 20. The phage was eluted by 100 mM triethylamine solution and maintained with 10 ml of mid-log phased XL1-Blue cells at 37 °C for 30 min, followed by shaking incubation for 30 min. Then, the XL1-Blue cells were cultured overnight at 30 °C in 2xYT agar plate containing 34 ug/ml of chloramphenicol and 1% glucose. After the fourth biopanning process, the binding activity to cMet was evaluated.

A microplate coated with cMet was incubated with 2% skim milk/PBS at 37 °C for 2 h and then washed with PBS. The microplate was treated with the phages obtained for each panning at room temperature for 1 h. After washing with PBS, horseradish peroxidase (HRP)-conjugated mouse anti-M13 antibody (GE Healthcare, USA) was treated at room temperature for 1 h. TMB solution (BD Biosciences, USA) was added to each well and absorbance was measured at 450 nm. As a result, among the 11 candidates which confirmed to have cMet binding activity, we selected VM507 as this candidate had the highest binding activity to cMet (data not shown). The phage display library screening and generation of IgG1 antibody were done by PharmAbcine (Daejeon, Korea).

### Characterization of VM507 antibody

The affinity of VM507 to its target cMet was analyzed using BIAcore (GE healthcare), according to the manufacturer’s instruction. cMet protein from human and mouse species were coated on the CM5 chip and induced the interaction between VM507 and cMet protein. Affinity value (Kd) was calculated based on the measurement of Ka and Kd (Table S1).

To analyze the target specificity of VM507, we performed microarray test using human protein Chip HuProt v3.1 (CDI Laboratories, Inc., USA), according to the manufacturer’s instruction.

We further analyzed the *in vivo* kinetic profile and biodistribution pattern of VM507 antibody. The antibody was injected to BALB/c background nude mice and its persistency was analyzed by measuring the level of VM507 in the mouse serum, using an ELISA specific to VM507. At the same time, major organs including the liver and spleen were prepared and the levels of VM507 in these organs were analyzed.

### Animals

Male C57BL/6 mice weighing 20 g were purchased from Sprague-Dawley (The Jackson Laboratory, Bar Harbor, ME). They were housed in the animal facilities at the Seoul National University Boramae Medical Center with free access to food and water. Unilateral ureteral obstruction (UUO) was performed using an established procedure. Briefly, mice under general anesthesia were subjected to complete ureteral obstruction by double-ligating the left ureter using 4–0 silk after exposure via a flack incision. Sham-operated mice had their ureters exposed and manipulated but not ligated^42,43^. Mice were randomly assigned into three groups (*n  M*6): (1) sham normal control; (2) UUO; (3) UUO receiving cMet agonistic Ab. cMet Ab was injected via tail vein four times (D-1, D b 1, D , 3, D , 7) at a dose of 20 mg/kg (300 μl, PBS) per injection.

### Histologic examination

Kidneys fixed in 4% paraformaldehyde and embedded in paraffin were sectioned at a 4-μm thickness and stained. Sections were deparaffinized in xylene and rehydrated in a graded series of ethanol. Endogenous peroxidase activity was blocked with 0.3% hydrogen peroxidase in methanol for 30 minutes at room temperature. Sections were microwaved for 30 minutes in an antigen unmasking solution to retrieve the antigen. After the sections were incubated with 10% goat serum for 1 hour at room temperature, they were incubated at 4 °C overnight with anti-Met (cMet) antibody (Abcam, Cambridge, UK) and anti-Met (cMet) (phospho Y1349) antibody (Abcam). The tissue sections were washed several times in PBS and incubated for 40 minutes with appropriate Alexa Fluor-conjugated secondary antibodies (Molecular Probes, Eugene, OR). 4′,6-Diamidino-2-phenylindole (DAPI; Molecular Probes) was used for counterstaining. For negative controls, the primary antibodies were omitted during the procedure. Confocal microscopy was performed with a Leica TCS SP8 STED CW (Leica, Wetzlar Germany).

### Cell culture of primary PTECs

We isolated PTECs from normal adjacent kidney tissue specimens from patients with renal cell carcinoma according to guidelines approved by the Institutional Review Board of Seoul National University Hospital (IRB no. 1506-097-681). After dissecting the cortex, the unaffected specimens were minced and digested with Hank’s balanced salt solution (HBSS) containing 3 mg/mL collagenase (Sigma-Aldrich, St. Louis, MO, USA). After centrifugation for 5 minutes at 500 g, cortical tubular cells were isolated. The cells were then incubated in DMEM/F12. After 4 hours of incubation, the tubules were collected and cultured on collagen-coated petri dishes (BD Biosciences, Franklin Lakes, NJ, USA) until colonies of epithelial cells were established, and 2 ± 3 passages were used in the current study. After 3 days of culture, the cells were detached from the dishes with a 3 mM EDTA solution and a minimal amount of trypsin. Cells (2 × 10^5^/well) were seeded on 8-well chamber slides in serum-free medium for 24 hours and then washed twice with PBS. Next, recombinant TGFβ (R&D Systems, Minneapolis, MN, USA) was added (final concentration, 10 ng/ml) except where indicated. Cells were incubated in the presence or absence of either recombinant HGF (10 and 20 mg/ml) or agonistic cMet antibody (250, 500 and 1000 ng/ml) for 48 hours. rHGF and cMet Ab were treated simultaneously with rTGFβ.

### Migration assay

HUVEC (CC-2517; Lonza, USA) migration was evaluated by the modified Boyden chamber assay. In all, 24-well transwell inserts (Corning, Corning, NY, USA) with porous polycarbonate filters (8-µm pore size) were coated with 1% gelatin in PBS. HUVECs suspended in M199 or Dulbecco’s modified Eagle’s medium supplemented with 1 or 3% FBS were added to the inserts at 4 × 10^4^ or 1 × 10^4^ cells per well. Recombinant HGF protein (RnD systems) was diluted to 50 ng/ml, and cMet Ab was diluted to 90 ng/ml in M199 or Dulbecco’s modified Eagle’s medium supplemented with 1 and 3% FBS, respectively; 600 µl of the final dilution was placed in the lower chamber. Considering the molecular weight, the concentration of cMet Ab was calculated as 90 ng/ml, which is comparable to 50 ng/ml recombinant HGF protein. HUVECs were allowed to migrate for 2 or 3 h at 37 °C in an incubator. The filters were then rinsed with PBS, fixed with 4% formaldehyde for 10 minutes and stained with 0.2% crystal violet. Migration was quantified by counting the positively stained cells in five separate high-power fields (X200) per well with Image-Pro Plus software (Media Cybernetics, Bethesda, MD, USA).

### Wound healing assay

PTECs from the 2nd or 3rd passage were seeded into a 96-well plate (5 × 10^4^ cells/well) in medium supplemented with 10% FBS and left to grow into a confluent monolayer. To assay the basal migratory ability of PTECs, the confluent cell monolayer was serum-starved for 12 hours, after which a wound was scratched on the monolayer using a p20 pipette tip. The cell-free area was measured immediately (0 hours) and 30 hours after the scratch. To study the PTEC response to cMet Ab and rHGF treatment, we cultured cells in medium supplemented with 10% FBS for 48 hours and then serum-starved the cells for 6 hours. The cells were subsequently treated with DMSO (control), cMet Ab (500 ng/mL), or rHGF (50 ng/mL). To visually assess viability, DAPI (Molecular Probes) was added, and cells were observed with a high-content imaging device (Operetta CLS; Perkin-Elmer, Germany) at different time points following treatment^17^.

### Real-time quantitative PCR analysis

Total RNA was extracted from PTECs, and the mRNA levels of the target genes were assayed by real-time quantitative PCR. Briefly, total RNA was isolated from PTECs using an RNeasy kit (Qiagen GmbH, Germany), and 500 ng of total RNA was reverse-transcribed using oligo-d(T) primers and AMV-RT Taq polymerase (Promega, WI, USA). Real-time qPCR was conducted on an ABI PRISM 7500 sequence detection system using either Assay-on-Demand TaqMan probes and primers (for E-cadherin, fibronectin, collagen 1, αSMA and GAPDH) or the SYBR Green method (for GAPDH, TNF-α, IFN-γ, IL-6 primer sequences are available in Table 1) (Applied Biosystems, CA, USA). Relative quantification was performed using the 2^−∆∆CT^ method. GAPDH served as a loading control^44^. All experiments were completed in triplicate.

**Table 1:** Sequences of real time PCR primers.

Target names	Species	Sense (5′-3′)	Antisense
GAPDH	MS	5′-TATGTCGTGGAGTCTACTGGT-3′	5′-GAGTTGTCATATTTCTCGT-3′
TNF-α	MS	5′-GGGACAAGGCTGCCCCGACT-3′	5′-TCCTTGGGGCAGGGGCTCTT-3′
IFN-γ	MS	5′-AACGCTACACACTGCATCTTGG-3′	5′-GCCGTGGCAGTAACAGCC-3′
IL-6	MS	5′-GTGCTCCTGGTATTGCTGGT-3′	5′-GGCTCCTCGTTTTCCTTCTT-3′

### Western blot analysis

PTECs were lysed in RIPA buffer [50 mM Tris·HCl, pH 7.3; 150 mM NaCl; 0.1 mM EDTA; 1% (vol/vol) sodium deoxycholate; 1% (vol/vol) Triton X-100; 0.2% NaF; and 100 mM Na3VO4] supplemented with complete protease inhibitors (Roche Applied Science, Indianapolis, IN). The kidney homogenate was centrifuged at 12,000 × *g* for 30 minutes at 4 °C, and the protein concentration of the supernatant was determined by the Bradford method. Different amounts of extracted protein were separated by SDS-PAGE and transferred onto Immobilon-FL 0.4-μm polyvinylidene difluoride membranes (Millipore). Tris-buffered saline containing 0.1% Tween 20 was used as the washing buffer. After probing with primary antibodies, anti-rabbit (1:5,000; Cell Signaling Technology, Danvers, MA) and anti-mouse (1:6,000 for β-actin; Cell Signaling Technology) antibodies were used as secondary antibodies. Detection of labeled proteins was performed with an enhanced chemiluminescence system (ECLTM PRN 2106; Amersham Pharmacia Biotech, Buckinghamshire, UK). The band intensities were analyzed using a gel documentation system (Bio-Rad Gel Doc 1000 and Multi-Analyst version 1.1)^17^.

Western immunoblotting was performed using primary antibodies against collagen 1 (Abcam), fibronectin (Santa Cruz Biotechnology, Dallas, TX), αSMA (Abcam), and β-actin (Cell Signaling Technology).

### Confocal microscopic examination

Confocal laser scanning images were acquired using a Leica 20X/0.7 NA objective lens an a DMI6000 inverted microscope equipped in the Leica TCS SP8 STED CW system. Nuclei were labeled with DAPI (colored blue) and excited by a Diode 405 UV laser. Antibodies conjugated to Alexa 488 dye (green fluorescence) were excited using an Argon 488 laser with 20% output and 5% excitation power, whereas antibodies conjugated to Alexa 555 dye (red fluorescence) were excited using a DPSS 561 laser with 10% excitation power. Emission wavelength spectra were fixed to 420–480 for DAPI, 500–550 for green fluorescence and 570–620 for red fluorescence using an Acousto-Optical beam splitter (AOBS). For signal amplification, a photomultiplier (PMT) detector was used with 800 detector gain without adjustment of background for green fluorescence, red fluorescence or deep red fluorescence. The DAPI signal was amplified using a Hybrid detector (HyD, Hamamatsu) with a detector gain of 100 detector. The scan speed was 400 Hz using a 4X line average with 1 Airy unit (66.57 µm) pinhole setting. Images were acquired using the sequential scanning mode of the LAS-X program with a 1024 × 1024 XY image format and 0.568 µm pixel size.

### Fluorescence-activated cell sorting (FACS) analysis

FACS analysis was performed using a BD FACSDiva instrument (version 8.0, BD Biosciences) to verify that the cMet Ab could bind cMet. A549 cells (ATCC) were used. Cells were cultured using F12 HAMS media supplemented with 10% FBS (Gibco), 1% P/X, 2% HEPES and EGM (Lonza, CC-3124). For the experiment, A549 cells (5 × 10^5^) were added to a 1.5 ml tube, and the culture was washed out. Then, 4 µl of cMet Ab (Abcam) was diluted in 100 µl of PBS, after which this solution was reacted with the cell suspension at 4 °C for 30 minutes. Afterward, the cells were centrifuged at 4000 rpm for 3 minutes and washed twice. Then, 2 µl of anti-human IgG-PE (Southern Biotech) was added to the suspension, and the cells were washed, resuspended in 1 ml of PBS, and then subjected to FACS analysis.

### Cell proliferation

A549 cells were used to investigate the proliferation effect of HGF. A549 cells were plated on a 96-well plate at 5000 cells/well. The next day, recombinant HGF protein (R&D systems) and cMet Ab were added to each well at concentrations of 50 ng/ml and 400 ng/ml, respectively. At 48 hours after treatment, the XTT assay (Roche) was used according to the manufacturer’s instructions. XTT labeling reagent and electron coupling reagent were added to the wells. After 4 hours, the OD (492–650 nm) was measured, and the results were analyzed.

### Statistical analysis

Animals were randomly assigned to control and treatment groups. All data examined were expressed as the mean ± SEM and were compared using Student’s t-tests or one-way ANOVA. All statistical analyses were performed using SPSS version 22 (IBM, Chicago, Illinois, United States). A *P* value less than 0.05 indicated statistical significance. The antibodies used in this study are listed in Table 2.

**Table 2:** Antibodies used in immunohistochemistry, immunofluorescence staining and immunoblotting.

Figure in the text	Antibody (vendors)	Catalog number
1A	anti-Met (cMet) Ab (Abcam)	ab59884-100
1B	DAPI (Molecular Probes)	D1306
	anti-Met (cMet) Ab (Abcam)	ab59884-100
	anti-aquaporin-1 Ab (Abcam)	ab15080
	Alexa Fluor-conjugated secondary Ab (Molecular Probes).	
	goat anti-mouse 488 (green)	A11001
	goat anti-mouse 555 (red)	A21422
	goat anti-rabbit 488	A11008
	goat anti-rabbit 555	A21428
	anti-Met (cMet) (phospho Y1349) Ab (Abcam).	ab68141
1C	rHGF (R&D Systems)	294-HG-005
2A–E	cMet Ab (Abcam)	ab59884-100
	Anti-Hunan IgG Ab (Abcam)	ab200699
	Anti-Human HGF R/cMet PE-conjugated Ab	FAB3582P
3A–H	Anti-Hunan IgG Ab (Abcam)	ab200699
	cMet Ab (Abcam)	ab59884-100
	DAPI (Molecular Probes)	D1306
4B	anti-Met (cMet) Ab (Abcam)	ab59884-100
	anti-aquaporin-1 Ab (Abcam)	ab15080
	anti-rabbit (Cell Signaling Technology) secondary	7074 s
4C	anti-mouse (Cell Signaling Technology) Secondary	7076 s
	anti-collagen 1 Ab (Abcam)	Ab90395
	anti-fibronectin Ab (Santa Cruz Biotechnology)	sc-8422
	anti-αSMA Ab (Abcam)	ab7817
	anti-beta-actin Ab (Cell Signaling Technology)	#4967
	anti-Met (cMet) (phospho Y1349) Ab (Abcam).	ab68141
5A	anti-Met (cMet) Ab (Abcam)	ab59884-100
	anti-beta-actin Ab (Cell Signaling Technology)	#4967
	DAPI (Molecular Probes)	D1306
6A	anti-fibronectin Ab (Santa Cruz Biotechnology)	sc-8422
	anti-collagen 4 Ab (Abcam)	ab21295
	anti-fibronectin Ab (Santa Cruz Biotechnology)	sc-8422
6B	anti-αSMA Ab (Abcam)	ab7817
	anti Bax Ab (Santa Cruz Biotechnology)	sc-7480
	anti-Bcl-2 Ab (Santa Cruz Biotechnology)	sc-7382
	anti-Met (cMet) (phospho Y1349) Ab (Abcam).	ab68141
	anti-Met (cMet) Ab (Abcam)	ab59884-100
	anti-beta-actin Ab (Cell Signaling Technology)	#4967
6D	DAPI (Molecular Probes)	D1306

HRP; horseradish peroxidase, IHC; immunohistochemistry, IF; immunofluorescence, DAPI; 4′,6-Diamidino-2-Phenylindole, Dihydrochloride, PTECs; proximal tubular epithelial cells, rTGFβ; recombinant transforming growth factor β, rHGF; recombinant hepatocyte growth factor, FACS; fluorescence-assisted cell sorting.

### Ethics statement

The present study was conducted with the approval of the Research Ethics Committee of the Seoul National University Boramae Medical Center. All procedures were performed in accordance with the ethical standards of the institutional and/or national research committee and with the 1964 Declaration of Helsinki and its later amendments or comparable ethical standards. Informed consent was obtained to human research participants for the use of tissue samples.

## Supplementary information

Supplementary information file

## Data Availability

The datasets generated during and/or analyzed during the current study are available from the corresponding author on reasonable request.
