# Supplementary material for: Renoprotective effects of a novel cMet agonistic antibody on kidney fibrosis
Source: Sci Rep. 2019 Sep 17;9:13495. doi: 10.1038/s41598-019-49756-z (PMC6749055; doi:10.1038/s41598-019-49756-z)
Supplement: Supplementary file 1 — Supplementary information file [file 41598_2019_49756_MOESM1_ESM.pdf]

Renoprotective effects of a novel cMet agonistic antibody on kidney fibrosis

Yong Chul Kim[1*], Junghun Lee[2*], Jung Nam An[3], Jin Hyuk Kim[3], Young-Wook Choi[3], Lilin Li[3,4], Sang Ho Kwon[2], Mi-Young Lee[2], Boeun Lee[2], Jae-Gyun Jeong[2], Seung-Shin Yu[2], Chun Soo Lim[3,4], Yon Su Kim[5,6], Sunyoung Kim[2], Seung Hee Yang[6,7], Jung Pyo Lee[3,4].

*Both authors contributed equally to this work.

Author Affiliations

[1]*Department of Internal Medicine, Seoul National University Hospital, Seoul, Korea.*

[2]*R&D Center for Innovative Medicines, ViroMed Co., Ltd., Seoul, Korea.*

[3]*Department of Internal Medicine, Seoul National University Boramae Medical Center, Seoul, Korea.*

[4]*Department of Internal Medicine, Seoul National University College of Medicine, Seoul, Korea.*

[5]*Department of Medical Science, Seoul National University College of Medicine, Seoul, Korea.*

[6]*Kidney Research Institute, Seoul National University College of Medicine, Seoul, Korea.*

[7]*Seoul National University Biomedical Research Institute, Seoul, Korea.*

Correspondence:

Seung Hee Yang, PhD

Associate Professor

Seoul National University Biomedical Research Institute and Kidney Research Institute, Seoul National University

101 Daehak-ro, Jongno-gu, Seoul, 03080, Republic of Korea

E-mail: ysh5794@gmail.com

and

Jung Pyo Lee, MD, PhD

Associate Professor

Department of Internal Medicine, Seoul National University Boramae Medical Center and Seoul National University College of Medicine

20 Boramae-ro 5-gil, Dongjak-gu, Seoul 07061, Republic of Korea.

E-mail: nephrolee@gmail.com

Supplementary Table S1.

	Control	CKD1	CKD3	CKD5
N (27)	6	9	6	5
Age	24.2 ± 5.9	34.8 ± 11.2	50.2 ± 8.4	50.8 ± 12.4
Sex (male, %)	4 (66.7)	3 (33.4)	3 (50.0)	3 (60.0)
eGFR	102.5 ± 5.0	94.2 ± 3.0	45.8 ± 4.5	11.9 ± 7.3

Supplementary Table S1. Baseline characteristics of IgA nephropathy patients.

Supplementary Table S2.

	Ka (1/Ms)	Kd (1/s)	KD (M)
Mouse cMet-Fc	2.58×10^{6}	1.59×10^{-3}	6.15×10^{-10}
Human cMet -Fc	2.71×10^{6}	8.07×10^{-4}	2.98×10^{-10}

Supplementary Table. S2. The binding affinity of VM507 to mouse and human cMet.

Supplementary Figure S1.

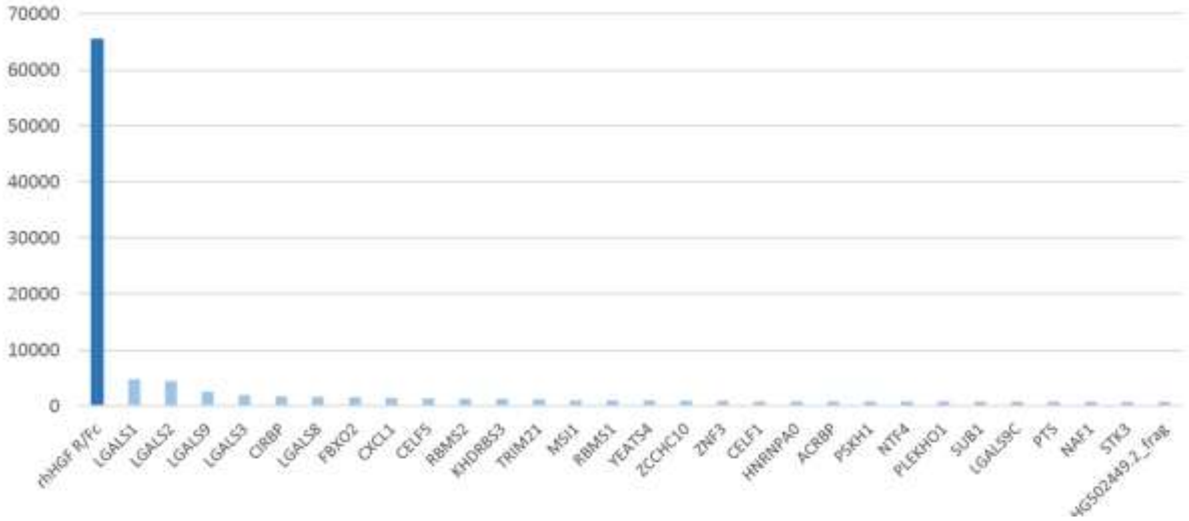

Supplementary Figure S1. Target specificity of VM507 by microarray test

Supplementary Figure S2.

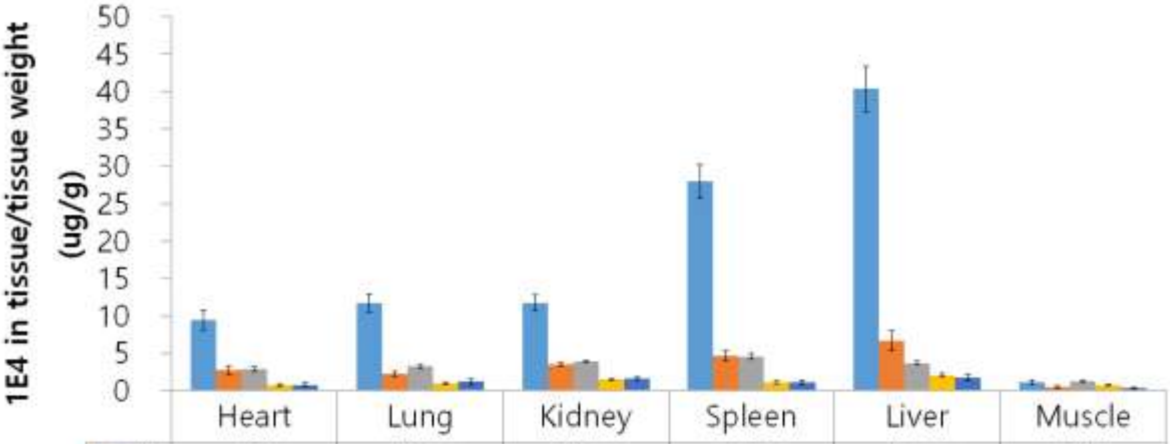

	Heart	Lung	Kidney	Spleen	Liver	Muscle
1H	9.43	11.69	11.69	27.98	40.36	1.16
8H	2.75	2.25	3.50	4.60	6.68	0.53
D.1	2.92	3.24	3.93	4.59	3.66	1.29
D.2	0.73	0.98	1.47	1.11	2.05	0.73
D.4	0.79	1.19	1.59	1.10	1.72	0.33

Supplementary Figure S2. In vivo Bio-distribution of VM507

Supplementary Figure S3.

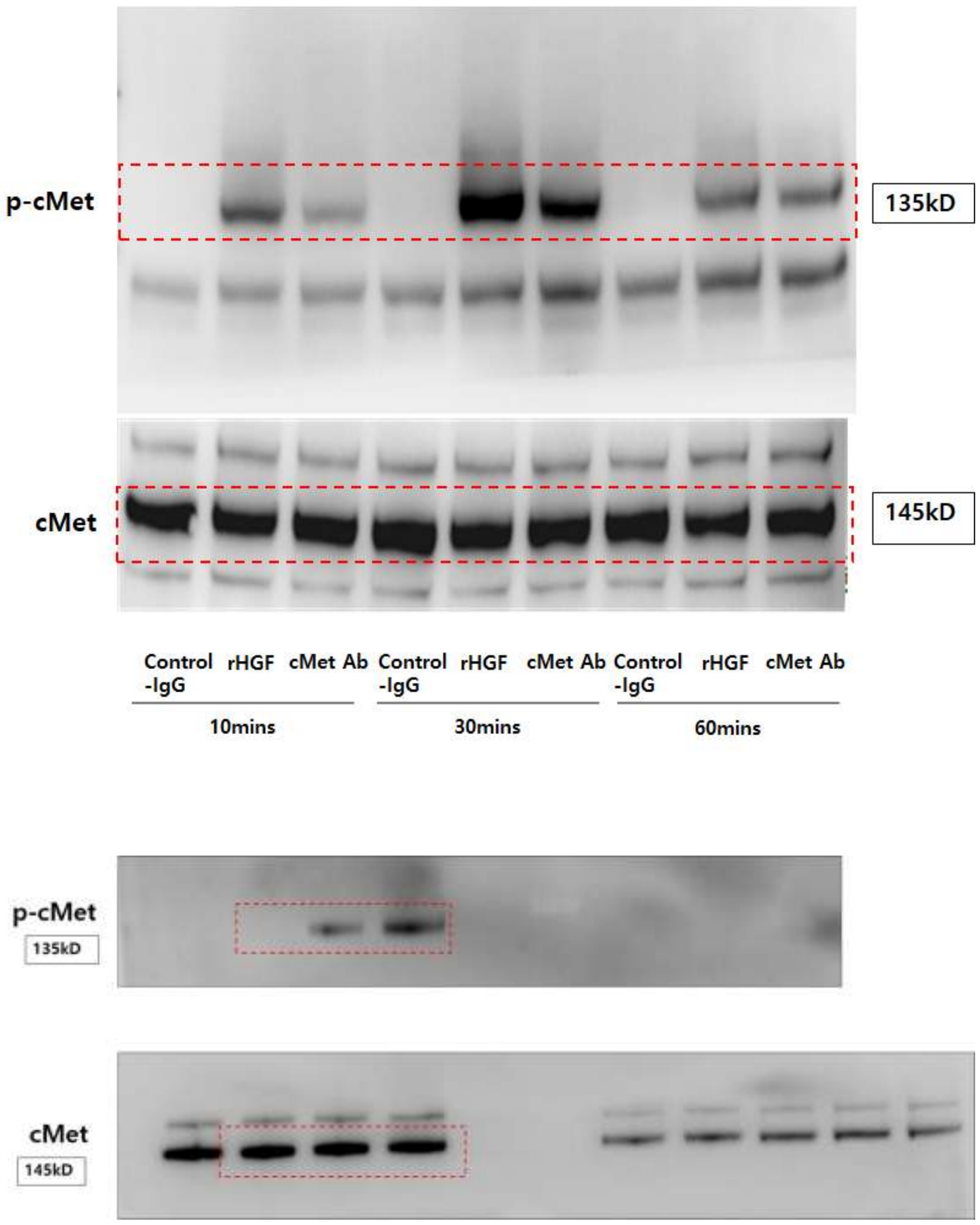

Supplementary Figure S3. Full length blots of Figure 2A & 2E. Red dotted lines show the cropping locations.

Supplementary Figure S4.

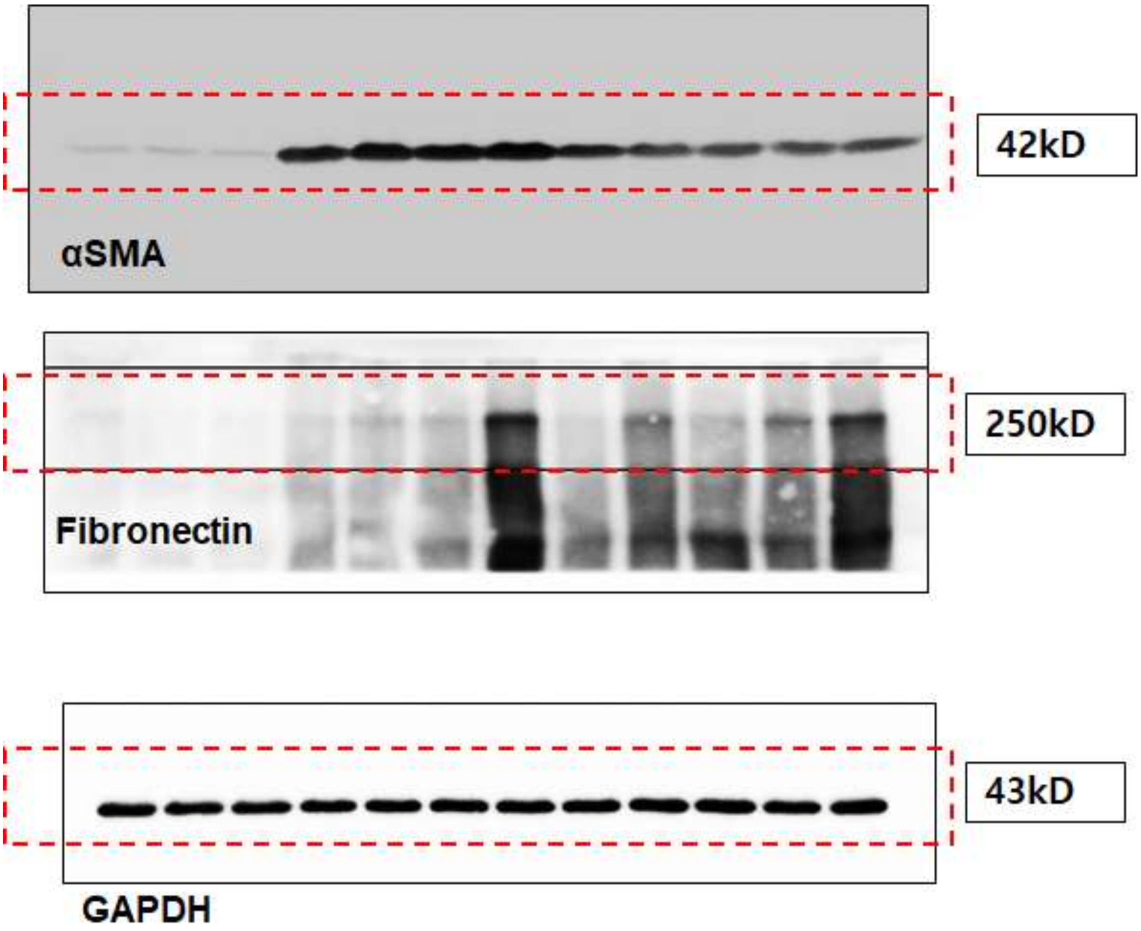
αSMA
42kD
Fibronectin
250kD
GAPDH
43kD

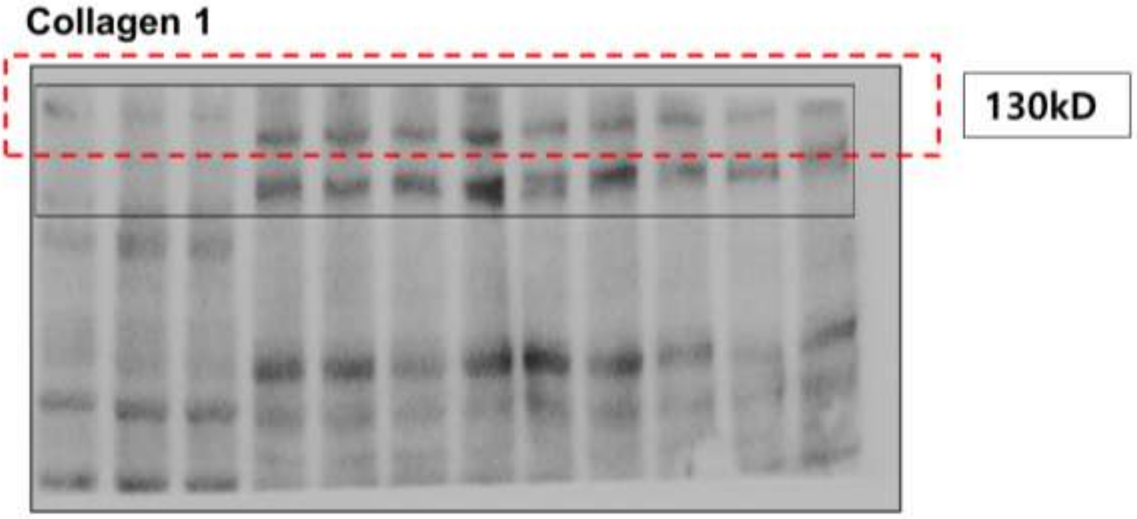

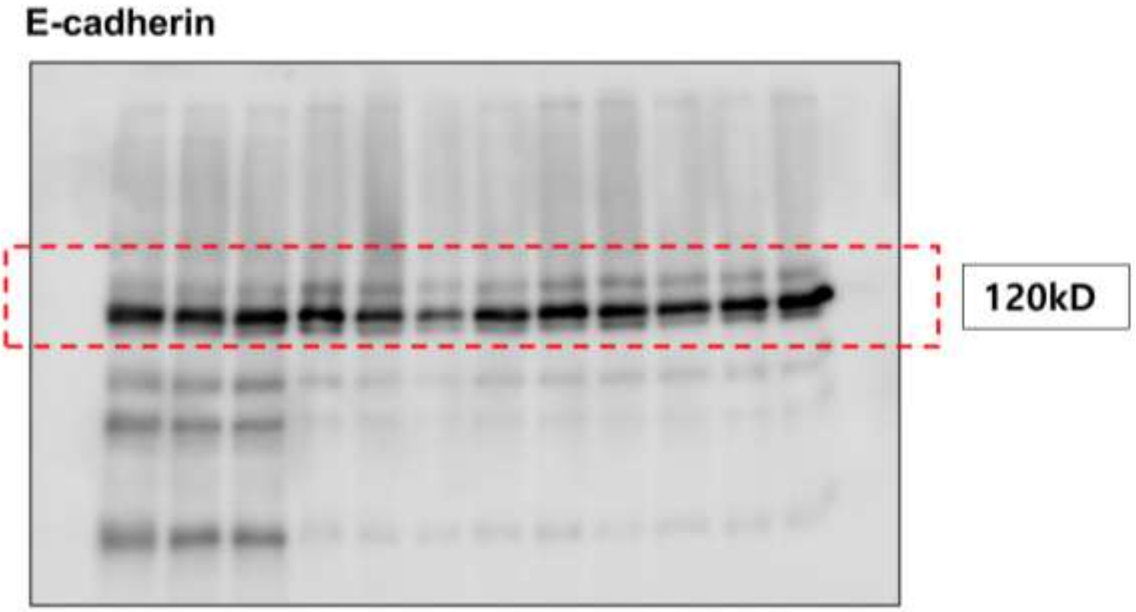

Supplementary Figure S4. Full length blots of Figure 4B. Red dotted lines show the cropping locations.

Supplementary Figure S5.

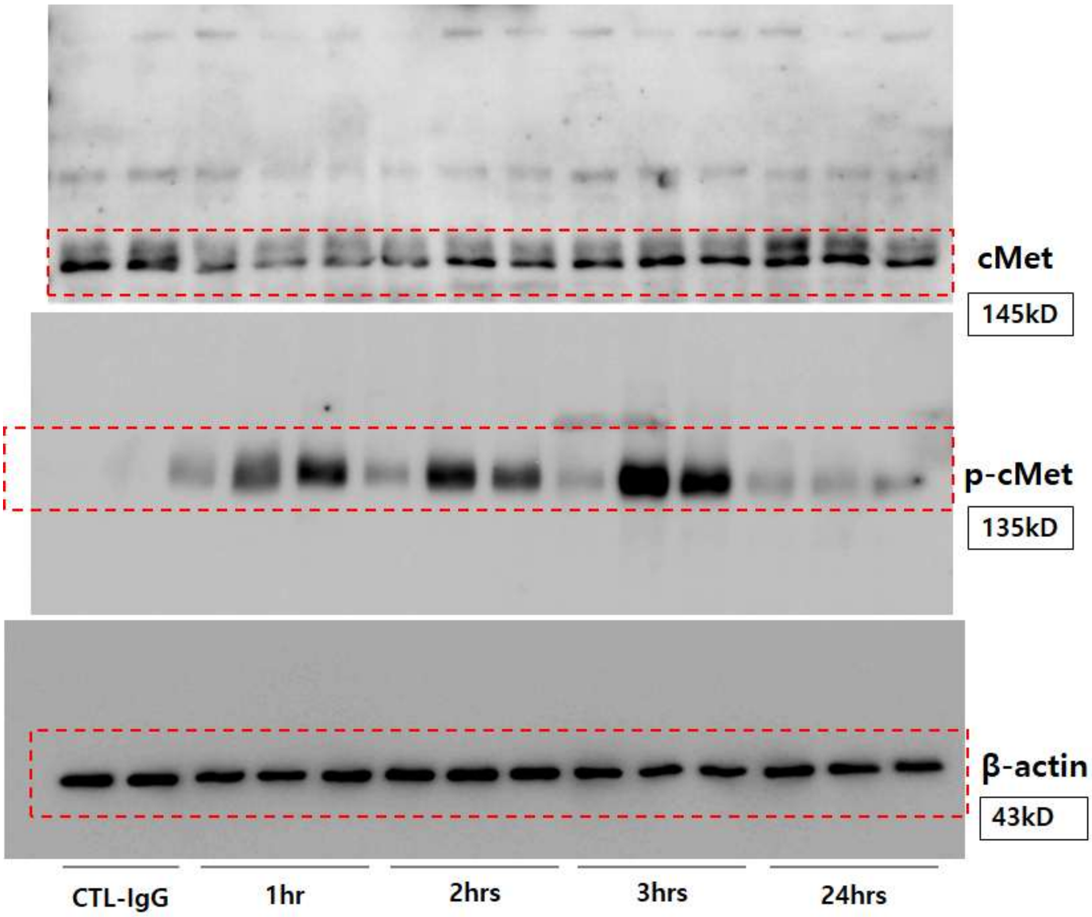

Supplementary Figure S5. Full length blots of Figure 5A. Red dotted lines show the cropping locations.

Supplementary Figure S6.

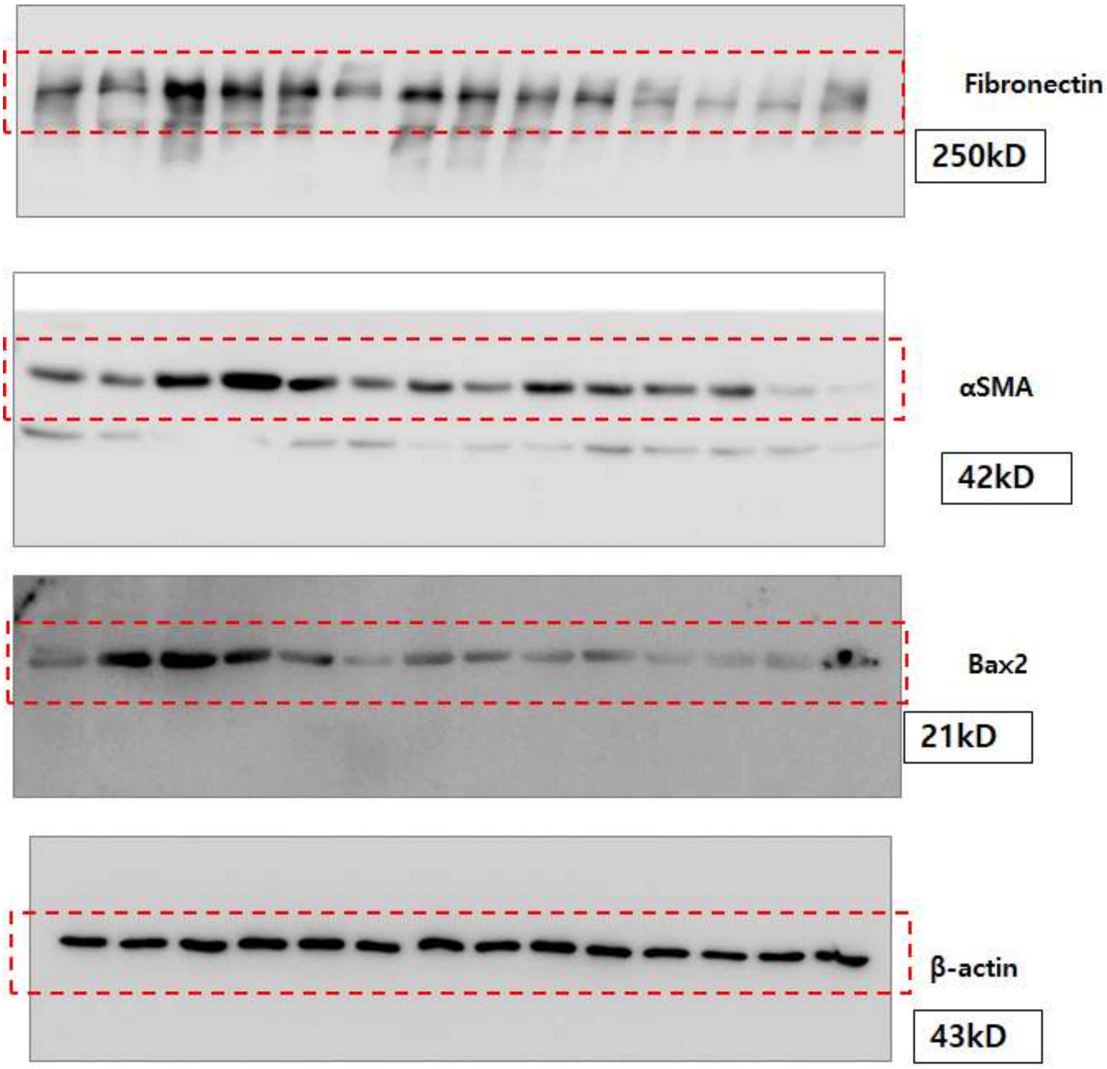

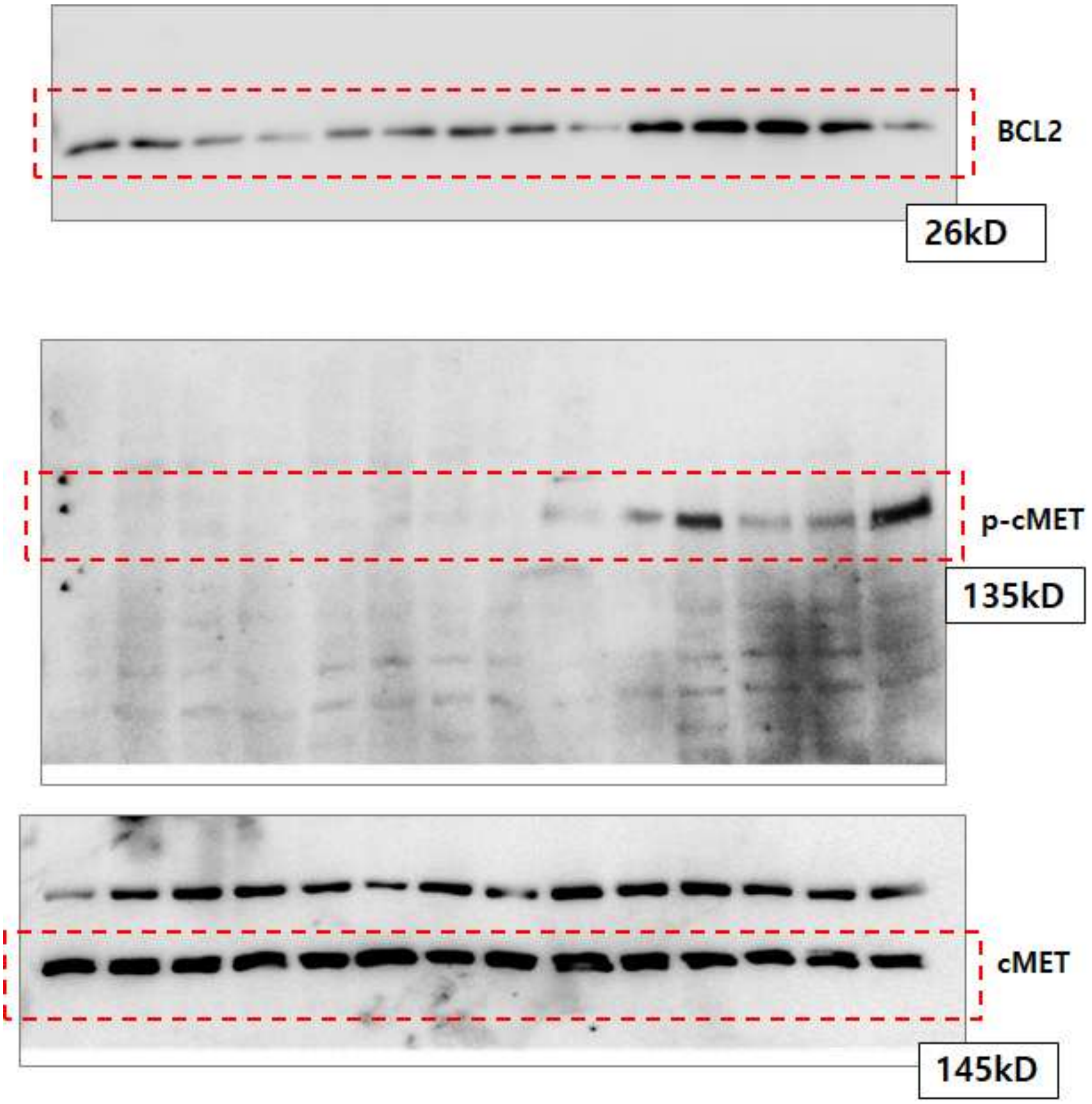

Supplementary Figure S6. Full length blots of Figure 6B. Red dotted lines show the cropping locations.
